# Inter-rater agreement and characterization of pleural line and subpleural fields in canine lung ultrasound: a comparative pilot study between high-frequency linear and curvilinear transducers using B- and M-mode ultrasonographic profiles

**DOI:** 10.1186/s13089-025-00401-z

**Published:** 2025-01-13

**Authors:** Kyle L. Granger, Liz Guieu, Søren R. Boysen

**Affiliations:** 1 Department of Clinical Sciences, James L. Voss Veterinary Teaching Hospital, College of Veterinary Medicine and Biomedical Sciences, Colorado State University, 300 W Drake Rd, Fort Collins, CO 80523 USA; 2 Department of Veterinary Clinical and Diagnostic Sciences, Faculty of Veterinary Medicine, University of Calgary, Calgary, AB Canada

**Keywords:** Lung ultrasound, Pleural line, Subpleural field, High-frequency linear transducer, Curvilinear transducer, Canine pulmonary disease

## Abstract

**Background:**

Lung ultrasound (LUS) is increasingly utilized in veterinary medicine to assess pulmonary conditions. However, the characterization of pleural line and subpleural fields using different ultrasound transducers, specifically high-frequency linear ultrasound transducers (HFLUT) and curvilinear transducers (CUT), remains underexplored in canine patients. This study aimed to evaluate inter-rater agreement in the characterization of pleural line and subpleural fields using B- and M-mode ultrasonography in dogs with and without respiratory distress.

**Results:**

Eighty-eight ultrasound clips from nine dogs were analyzed. HFLUT demonstrated strong inter-rater agreement in B-mode (κ = 0.89) and near-perfect agreement in M-mode (κ = 1.00) for pleural line homogeneity. In contrast, CUT showed minimal agreement in both B-mode (κ = 0.34) and M-mode (κ = 0.37). Homogeneous pleural lines were predominantly observed in control dogs or those with cardiogenic pulmonary edema (CPE), while non-homogeneous pleural lines were more common in dogs with non-cardiogenic alveolar-interstitial syndrome (NCAIS). Vertical subpleural fields identified in M-mode were associated with both CPE and NCAIS, whereas horizontal fields were more often observed in control dogs.

**Conclusions:**

HFLUT offers superior inter-rater reliability for characterizing pleural and subpleural features in canine LUS compared to CUT, particularly in M-mode. These findings suggest HFLUT may enhance diagnostic accuracy for pulmonary conditions in dogs. Further studies are needed to explore the diagnostic potential of LUS in differentiating vertical artifact (e.g., B-lines) etiologies in veterinary patients.

**Supplementary Information:**

The online version contains supplementary material available at 10.1186/s13089-025-00401-z.

## Background

In both human and veterinary medicine, point-of-care ultrasound (POCUS) has emerged as a valuable patient-side diagnostic tool, offering clinicians non-invasive insights into various pathological conditions, particularly in the realm of pulmonary parenchymal disease.

In veterinary medicine, lung POCUS is predominantly performed using convex or curvilinear transducers (CUTs) in “B-mode,”—the two-dimensional ultrasound imaging mode that provides detailed structural information of the scanned area [11, 21, 22]. One of the aims of our LUS study is to identify vertical artifacts, defined as echogenic lines extending from the pleural line, moving synchronously with it, erasing A-lines, and extending to the far-field of the image [11, 21, 22]. While the presence of fewer than three vertical artifacts may indicate healthy lungs, micro-atelectasis, or interlobular structures in control animals [11, 25], the presence of three or more vertical artifacts generally suggests non-specific alveolar-interstitial syndrome [12, 22]. This condition has a wide range of differential diagnoses, including cardiogenic pulmonary edema (CPE) and non-cardiogenic alveolar-interstitial syndrome (NCAIS). In people, the characteristics of the pleural line—such as irregularity, thickening, or discontinuity—provide additional insights into underlying pulmonary pathologies like pneumonia, pulmonary fibrosis, and acute respiratory distress syndrome (ARDS) [22, 23, 29, 33]. These observations suggest that detailed ultrasonographic examination of pleural line characteristics could significantly enhance diagnostic precision and facilitate earlier treatment in the clinical setting, a concept which has yet to be explored in veterinary medicine.

While B-mode ultrasound has proven effective in detecting gross pulmonary abnormalities, it may lack the resolution required to visualize subtle changes in the pleural line and subpleural field, which may be indicators of underlying lung pathology [8]. In contrast, M-mode ultrasound, also known as “motion” mode, offers clinicians the ability to visualize dynamic movement of the lung surface and in some cases structures within the lung, including the motion of the pleura and underlying pulmonary parenchyma. In human medicine M-mode characteristics of the pleural line and subpleural lung parenchyma have been used to help differentiate between CPE and NCAIS. Additionally, the subpleural pattern, formed in part by acoustic traps identified through M-mode imaging, may further aid in the diagnosis of sonographic lung interstitial syndrome (SLIS), with specific fields correlating with different pathological conditions [18–20]. These applications of ultrasound highlight the potential of ultrasound imaging in facilitating rapid and accurate diagnosis, particularly in emergency settings.

In human medicine, various ultrasound transducers have been evaluated in lung POCUS [3, 9, 13]. The general concensus is to use high-frequency linear transducers (HFLUTs) for lung POCUS given their higher resolution, allowing better visualization (and interpretation) of the pleural line and superficial pulmonary parenchymal detail [2, 9, 17, 28]. Prior veterinary studies have predominantly focused on using CUTs for lung POCUS [10, 35, 37], while only a few studies that have utilized HFLUTs. These studies largely focused on the interobserver agreement among interpreters for pulmonary parenchymal disease using HFLUTs and CUTs [34, 37]. Conversely, veterinary studies centered on the ultrasonographic assessment of the pleural line using either transducer remains scarce [26, 37], with no studies evaluating the utility of M-mode imaging. Currently, veterinary medicine lacks guidelines regarding the utility and potential benefit of one transducer (CUT versus HFLUT) over the other for evaluation of pulmonary parenchymal disease in dogs and cats.

Our objectives were to (1) evaluate inter-rater agreement of 2 interpreters to characterize pleural line and subpleural field of B- and M-mode ultrasound clips obtained from dogs without evidence of cardiopulmonary disease on examination, henceforth referred to as “control(s)”, and dogs presenting in respiratory distress using a HFLUT and CUT; and (2) describe the ultrasonographic characteristics of the pleural line and subpleural field in control dogs, dogs with CPE, and dogs with NCAIS in both B-mode and M-mode using a HFLUT and CUT. We hypothesized that when assessing pleural line and subpleural field characteristics of clips obtained from dogs presenting in respiratory distress using B- and M-mode ultrasonography, the HFLUT would exhibit higher inter-rater agreement than the CUT. We also hypothesized that the ultrasonographic characteristics of the pleural line and subpleural field in dogs with CPE and NCAIS would differ from those in control dogs.

## Materials and methods

Procedures were approved by the Institutional Animal Care and Use Committee of the College of Veterinary Medicine at Colorado State University. Informed owner consent was attained for each patient enrolled in the study.

### Subject selection

In each patient, basic demographic and medical data were recorded (species, breed, gender, age, body weight, history of disease, relevant medications given at admission). A complete clinical examination (including heart and respiratory rates, presence of heart murmur and abnormal respiratory sounds) and thoracic POCUS was performed by one of the investigators (KG). On this basis, an initial diagnosis was made, and empiric therapy started at the attending clinician’s discretion.

Dogs enrolled were assigned to the control or study group based on the absence or presence of pulmonary edema (PE), respectively, on diagnostic imaging (DI). Diagnostic imaging included at least one of the following in conjunction with thoracic POCUS: thoracic radiographs (TXR; 2-views minimum) reviewed by a veterinary radiology resident or diplomate of the American College of Veterinary Radiology, and/or echocardiography performed by a veterinary cardiology resident or diplomate of the American College of Internal Medicine subspecialty Cardiology. The latter was required to differentiate, and further characterize, PE as either CPE or NCAIS.

Dogs were included in the control group if they had no evidence of cardiopulmonary disease based on history and physical examination as well as absence of abnormalities on thoracic POCUS and thoracic radiographs. Exclusion criteria included recent diagnosis of cardiopulmonary disease, presence of a heart murmur on cardiac auscultation, and/or evidence of pleural effusion, vertical artifacts, or enlarged LA:Ao on thoracic POCUS.

Dogs were allocated to the CPE study group if they had vertical artifacts on thoracic POCUS and met one or more of the following criteria:

1. An enlarged LA:Ao of ≥ 1.7:1 on POCUS, and a positive response to furosemide (defined as a decrease in respiratory rate by 10 breaths per minute).
2. Radiographic evidence of left atrial enlargement, cardiomegaly and pulmonary vessels distention, with a final clinical diagnosis of congestive heart failure.
3. Echocardiographic evidence of left atrial enlargement as determined by a board-certified cardiologist or cardiology resident

Dogs were allocated to the NCAIS study group if they had vertical artifacts on thoracic POCUS and met one or more of the following criteria:

1. No evidence of LA:Ao enlargement on POCUS
2. Absence of echocardiographic abnormalities consistent with primary cardiac disease as determined by a board certified cardiologist or cardiology resident
3. Radiographic evidence of NCAIS without signs of congestive heart failure (i.e., absence of left atrial enlargement, cardiomegaly or pulmonary veins distention).

Exclusion criteria for both PE subgroups included evidence of both CPE and NCAIS (i.e., left-sided congestive heart failure and pulmonary hypertension), and/or non-effusive pleural space diseases including pneumothorax, diaphragmatic herniation, and/or neoplasia, as diagnosed via DI. Pleural effusion was not an exclusion criteria unless it was deemed to be non-cardiogenic in origin and/or the effusion, if sampled, did not align with established parameters for a modified transudate.

### Transthoracic ultrasonographic imaging

#### Transthoracic ultrasound scanning technique

All subjects were scanned using a standardized protocol with a C11 × 8–5 MHz conventional curvilinear/convex transducer (CUT; Sonosite II, Fujifilm^®^) and an HFL38xi 13–6 MHz high-frequency linear transducer (HFLUT; Sonosite II, Fujifilm^®^). The subjects were scanned in either a sitting, standing, or sternal recumbency position with the transducer in the transverse plane, perpendicular to the thoracic wall surface, and adjusted to scan through the intercostal space. For lung ultrasound with the HFLUT, the center frequency selected is 4.82 MHz, supporting a frame rate of 1312 Hz, which provides a standard imaging quality for pulmonary assessments. The maximum mechanical index for this transducer is set to 1.5, enabling adequate penetration while minimizing tissue impact. For the LUS, a single focal point was utilized, with the focal position determined by system presets. While the ideal focal setting for LUS is at or just beyond the pleural line, the specific focal position in this study was not adjusted to this standard. The SII system features automatic focal point adjustment, which dynamically optimizes the image resolution based on the target depth. This automation ensures precise visualization of specific areas within the lung field without requiring manual calibration, enhancing efficiency and consistency in clinical settings (FUJIFILM Sonosite [16]).

The ultrasound examination began in the dorsal third of the thorax. The ultrasound transducer was placed approximately over the 9th intercostal space (ICS), perpendicular to the ribs, and the “bat” sign was identified. If the Curtain Sign (an ultrasound feature marking the basal edge of the lung) was encountered, the transducer was slid cranially until it was situated over the lung. The transducer was then slid cranially, maintaining its transverse orientation, examining each intercostal space until reaching approximately the 4th ICS, where the front limb prevented further transducer advancement. Scanning proceeded with the middle third of the thorax, from cranial to caudal, until approximately the 6th ICS was encountered. The extent of scanning in this region is limited by the curtain sign, which typically marks the practical scanning boundary caudally. In the ventral third of the thorax, which considers the cardiac notch, the scan started around the 6th ICS (as dictated by the curtain sign, which represents the caudal border), and continued cranially to the 2nd or 3rd ICS, including the axillary region and lung surface just dorsal to the cardiac notch if the cardiac notch was encountered.

**Fig. 1:**
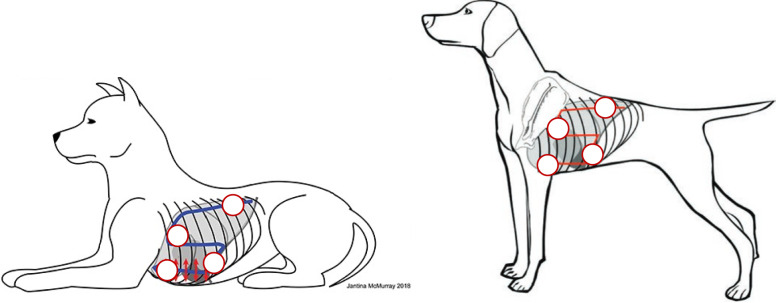
Pictographic Depiction of the Ultrasonographic Scanning Technique (modified Armenise) in Dogs with identification of the zones of interest. This figure illustrates the ultrasonographic technique employed to acquire images from dogs in both sternal recumbency and standing positions. The process begins at the upper caudal dorsal ninth intercostal space, with the ultrasound probe initially placed perpendicular to the ribs to identify the “bat” sign. The probe is then methodically moved cranially, maintaining a transverse orientation to examine each intercostal space up to the fourth. The scanning continues in the middle third of the thorax, moving caudally one intercostal space at a time from the fourth to the ninth intercostal spaces. The final scans cover the ventral third of the thorax, moving cranially from the 6th intercostal space back to the fourth. The red circles in each figure highlight the zones of interest along the scanning path. *Left image adapted from *[5]* and the image from the right is adapted from Boysen *et al*. 2023* [4]

The tomographic plane was adjusted to optimize the pleural line appearance and obtain a standardized clip in each area. Once an acceptable view was ascertained, the depth was adjusted between 2 and 6 cm to bring the pleural line to a central screen position, depending on the animal’s body condition. After the pleural line was centralized, a minimum of two six-second 2D (B-mode) clips were obtained and stored. Capture of clips using both B- and M-mode were attained from any of the following zones of interest (ZOI): the dorsal region: caudodorsal (caudal border of the 9th ICS), perihilar (caudal aspect of the 6th ICS) and in the ventral region: mediolateral (caudal border of the 4th ICS), and cranioventral (cranial border of the 4th ICS and the axilla) zones (Fig. 1). The M-mode scan line was placed perpendicularly to the pleural line and the image was stored. If the dog’s clinical status allowed, these clips were attained from each of the hemithoraxes for a minimum of 2 clips per patient (one from each hemithorax), provided the patient was sufficiently stable to obtain bilateral B- and M-mode clip captures. The clips were then exported for offline blinded analysis by the reviewers (LG and SB).

Adjustments to the scanning boundaries were based on individual variations in dog anatomy and respiratory status to ensure the protocol accurately reflects practical scanning limits, anatomical variances among different dogs, and prioritized the patient’s wellbeing.

#### Transthoracic ultrasound imaging definitions

**Fig. 2:**
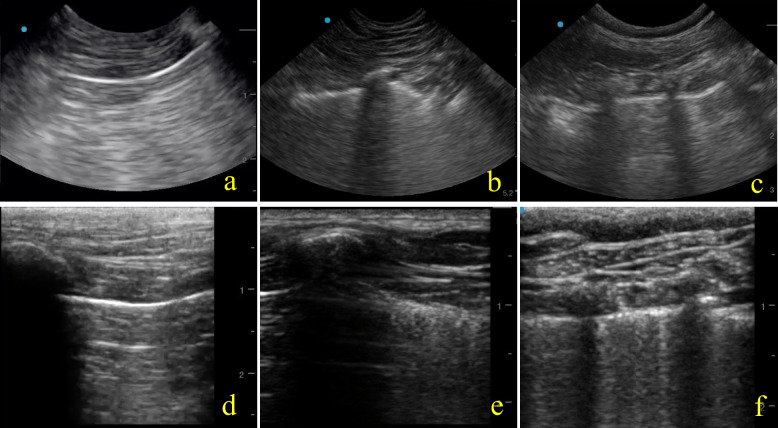
Comparative Ultrasound Imaging of Pleural Line and Subpleural Field in Dogs Using Curvilinear Ultrasound Transducers (CUT) and High-Frequency Linear Ultrasound Transducers (HFLUT). Panels A, B, and C show B-mode images captured using CUT. Panels D, E, and F show B-mode images captured using HFLUT. Panels A & D each shows a homogenous pleural line with A-lines in the subpleural field to create a horizontal subpleural field. Panels B & E each shows a non-homogenous, fragmented pleural line with lack of A-lines within the subpleural field creating a vertical subpleural field. Panels C & F each shows a non-homogenous, irregular pleural line with a lack of A-lines in the subpleural field creating a vertical subpleural field

**Fig. 3:**
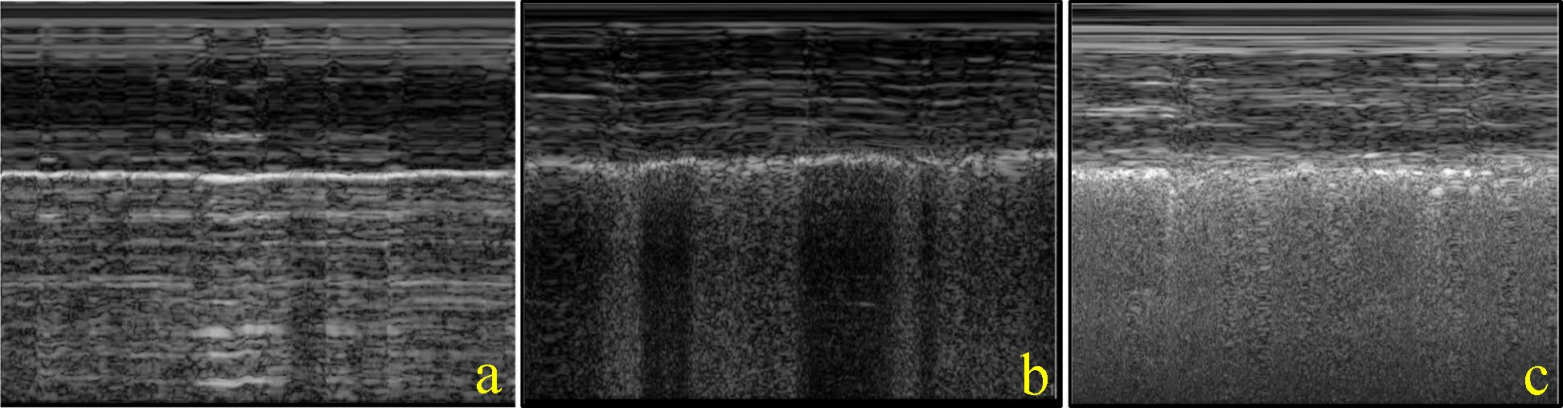
M-mode clips using a high-frequency linear ultrasound probe. **a** homogenous pleural line with A-lines in the subpleural field creating a horizontal field in the subpleural field. **b** Non-homogenous pleural line with subclassification of irregular with vertical artifacts extending from the pleural line to create a vertical subpleural field. **c** Non-homogenous pleural line with subclassification of fragmented with vertical artifacts extending from the pleural line through the subpleural field to create a vertical subpleural field

Given the lack of a concensus for the descriptions of the pleural line abnormalities within the human literature, the following definitions have been created by the authors and/or adapted from Fischer et al. [15] to better describe characteristics of pleural line abnormalities and subpleural fields within the veterinary medical field (Figs. 2 and 3).

- **Pleural line:** the echo-rich line at the junction of the visceral and the parietal pleura using either B- or M-mode ultrasonography
- **A-lines:** artifacts that are clearly visualized as horizontal reverberation artifacts of the echo-rich pleural line using either B- or M-mode ultrasonography [15]
- **Vertical artifacts:** well-defined, laserlike, vertical, echogenic lines arising from the pleural line, that erase A-lines from the subpleural field using either B- or M-mode ultrasonography [15]
- **Non-homogenous pleural line:** represents a pleural line which is heterogenous and disrupted which can be distinguished easily from a homogenous line in real time using either B- or M-mode ultrasonography [15]
- **Homogenous pleural line:** a continuous, echo-rich line without any evidence of irregularities or discontinuity using either B- or M-mode ultrasonography
  - **Irregular:** Pleural line exhibits variations in thickness with deviations of the line’s width using either B- or M-mode ultrasonography
  - **Fragmented:** Discontinuities of the pleural line length, appearing heterogeneous and non-linear on B-mode or M-mode ultrasonography, with each segment assessed within its respective field using either B- or M-mode ultrasonography
- **Vertical subpleural field:** lack of horizontal pleural reflection (or lack of “A” line artifacts) with concurrent increased echogenicity below the pleural line using M-mode ultrasonography
- **Horizontal subpleural field:** presence of the horizontal pleural reflection (or presence of A-lines) without evidence of increased echogenicity below the pleural line using M-mode ultrasonography

### Reviewer asssessment of B-mode and M-mode clips

To characterize the B- and M-mode, six second clips of the pleural line were captured for each patient in at least two ZOIs. All scans were captured by one investigator (KG) and subsequently reviewed by the remaining two investigators (SR, LG). The first investigator (KG) has a technical background with > 4 years of experience in lung POCUS. The remaining two investigators (SR, LG) have > 10 years of experience in lung POCUS. Clips were labeled with a randomly assigned patient identifier and reviewers were blinded to the patient and image ZOI to further evaluate interobserver variability and reliability.

#### One dimensional (M-mode) clip and two dimensional (B-mode) clip assessment

Using the aforementioned definitions, B-mode and M-mode clips were reviewed and assessed for pleural line homogeneity. Pleural lines were characterized as either (A) homogenous or (B) non-homogenous. If assessed as non-homogenous, the pleural line was further characterized as either irregular or fragmented. In M-mode clips, the subpleural field was also assessed and subsequently characterized as horizontal or vertical [32]. Our methodology did not involve quantitative counting of vertical artifacts but rather focused on qualitative characterization of pleural and subpleural fields to provide a robust assessment of lung pathology.

## Statistics

A sample size calculation was performed and found 9 clips to be a sufficient sample size for each group based on an area under the curve (AUC) of 0.85. Cohen’s Kappa statistics and 95% confidence limit were calculated to find the agreement between the rating from the 2 reviewers in each mode within HFLUT and CUT separately. Agreement could only be calculated for the parameters which had data in all the categories. The value of κ ranges from 0 to 1, where 0 indicates no agreement and values closer to 1 indicate stronger agreement. Specifically, a κ value from 0 to 0.20 signifies no agreement, from 0.21 to 0.39 indicates minimal agreement, from 0.40 to 0.59 is considered weak agreement, from 0.60 to 0.79 represents moderate agreement, from 0.80 to 0.90 corresponds to strong agreement, and a value above 0.90 is interpreted as near perfect agreement. This scale helps in quantifying the level of concurrence between reviewers, accounting for the possibility of agreement occurring by chance. Fischer’s exact test was used to analyze the pleural line and subpleural field characteristics to determine if there were significant differences between the groups. SAS v9.4 (SAS Institute Inc., Cary, NC) was used for all statistical analyses and a *p*-value of 0.05 was set as a criteria for statistical significance.

## Results

Between June 2023 and November 2023, a total of 25 dogs were assessed for potential inclusion in this study. 16 dogs were excluded due to the presence of more than a single disease process contributing to PE, including neoplasia and pleural effusion. The remaining nine dogs, consisting of five spayed females and four neutered males, were included. The sample population included 88 ultrasound media for analysis: 27 B-mode clips and 61 M-mode clips.

### Population demographics

Control Dogs (n = 3): Mixed breed dogs, median age of 5 years (range: 1–7).

NCAIS (n = 3): 1 Welsh Corgi, 1 French Bulldog, and 1 mixed breed, median age of 3 years (range: 1–6). Underlying causes of respiratory distress included self-induced suffocation by non-breathable bag, cluster seizures, and pulmonary hypertension.

CPE (n = 3): 1 Maltese, 1 Boxer, and 1 Yorkshire Terrier, median age of 10 years (range: 6–13). All were diagnosed with left-sided congestive heart failure.

### Ultrasound clip interrater agreement

#### HFLUT clips (Table 1)

**Table 1:** Classification of pleural line and subpleural field characteristics by disease group using the HFLUT

HFLUT General Study Population Composition				
Category	Control	CPE	NCAIS	Total Clips
Number of Dogs	3	3	3	–
Number of Clips (B-Mode & M-Mode)	16	12	24	52
HFLUT B-Mode Composition (n = 18)				
Category	Control	CPE	NCAIS	Total Clips
*Number of B-mode Clips*	*6*	*4*	*8*	*18*
Reviewer Discrepancies of Pleural Line Classification	1	0	0	1
Homogeneous Pleural Line	5	3	0	8
Non-Homogeneous Pleural Line	1	1	8	10
*Subclassification as Irregular*	*0*	*0*	*6*	*6*
*Subclassification as Fragmented*	*0*	*0*	*1*	*1*
*Reviewer Discrepancies of Pleural Line Subclassification*	*1*	*0*	*1*	*2*
HFLUT M-Mode Composition (n = 34)				
Category	Control	CPE	NCAIS	Total Clips
*Number of M-Mode Clips*	*10*	*8*	*16*	*34*
Reviewer Discrepancies of Pleural Line Classification	0	0	0	0
Homogeneous Pleural Line	10	8	0	18
Non-Homogeneous Pleural Line	0	0	16	16
*Subclassification as Irregular*	*0*	*0*	*7*	*7*
*Subclassification as Fragmented*	*0*	*0*	*7*	*7*
*Reviewer Discrepancies of Pleural Line Subclassification*	*0*	*0*	*2*	*2*
Horizontal Subpleural Field	10	2	4	16
Vertical Subpleural Field	0	6	12	18
Reviewer Discrepancies of Subpleural Field Characterization	0	0	2	2

1D One Dimensional, *B−mode* Brightness mode, *CPE* Cardiogenic Pulmonary Edema, *HFLUT* High frequency linear ultrasound transducer, *M−mode* Motion mode, *NCAIS* Non−Cardiogenic Alveolar−Interstitial Syndrome

There were 18 HFLUT clips assessed by the reviewers in B-mode. Overall, there was strong interobserver agreement (κ = 0.89, 95% CI 0.68–1) for pleural line classification (homogeneous (n = 8), non-homogeneous (n = 9), discrepancy (n = 1) [Control]). A moderate agreement among the reviewers (κ = 0.72, 95% CI 0.44–0.99) was identified for subclassification of non-homogeneous pleural lines (irregular (n = 6), fragmented (n = 1), discrepancies (n = 2) [1 in Control, 1 in NCAIS]).

There were 34 HFLUT clips assessed by the reviewers in M-mode. Overall, there was perfect interobserver agreement (κ = 1, 95% CI 1–1) for pleural line classification (homogeneous (n = 18), non-homogeneous (n = 16)). Almost perfect inter-rater agreement (κ = 0.90, 95% CI 0.78–1) was identified for subcategorization of the 16 non-homogeneous pleural lines (fragmented (n = 7), irregular (n = 7), discrepancies (n = 2) [2 in NCAIS]). A strong reviewer agreement (κ = 0.88, 95% CI 0.72–1) was observed for subpleural field field classification (horizontal (n = 16), vertical (n = 16), discrepancy (n = 2) [2 in NCAIS]).

#### CUT clips (Table 2)

**Table 2:** Classification of pleural line and subpleural field characteristics by disease group using the CUT

CUT General Study Population Composition				
Category	Control	CPE	NCAIS	Total Clips
Number of Dogs	3	3	3	–
Number of Clips (B-Mode & M-Mode)	12	13	11	36
CUT B-Mode Composition (n = 9)				
Category	Control	CPE	NCAIS	Total Clips
Number of Clips	3	3	3	9
Reviewer Discrepancies of Pleural Line Classification	0	0	3	3
Homogeneous Pleural Line	3	0	0	3
Non-Homogeneous Pleural Line	0	1	2	3
*Subclassification as Irregular*	0	0	1	1
*Subclassification as Fragmented*	0	0	0	0
*Reviewer Discrepancies of Pleural Line Subclassification*	0	0	3	3
CUT M-Mode Composition (n = 27)				
Category	Control	CPE	NCAIS	Total Clips
Number of Clips	9	10	8	27
Reviewer Discrepancies of Pleural Line Classification	4	2	2	8
Homogeneous Pleural Line	5	0	0	5
Non-Homogeneous Pleural Line	0	8	6	14
*Subclassification as Irregular*	0	3	2	5
*Subclassification as Fragmented*	0	2	2	4
*Reviewer Discrepancies of Pleural Line Subclassification*	0	3	2	5
Horizontal Subpleural Field	6	0	0	6
Vertical Subpleural Field	0	6	6	12
Reviewer Discrepancies of Subpleural Field Classification	3	4	2	9

*1D* One Dimensional, *B−mode* Brightness mode, *CPE* Cardiogenic Pulmonary Edema, *CUT* Curvilinear ultrasound transducer, *M−mode* Motion mode, *NCAIS* Non−Cardiogenic Alveolar−Interstitial Syndrome

There were 9 CUT clips assessed by the reviewers in B-mode. Overall, there was minimal agreement between reviewers (κ = 0.34) for pleural line classification (homogeneous (n = 3), non-homogeneous (n = 3), discrepancy (n = 3) [1 in CPE, 2 in NCAIS]). A weak agreement between reviewers (κ = 0.44, 95% CI – 0.02–0.89) was identified for the subclassification of a non-homogeneous pleural line (irregular (n = 1), fragmented (n = 0), discrepancies (n = 2) [2 in CPE]).

There were 27 CUT clips assessed by the reviewers in M-mode. Overall, there was minimal agreement between reviewers (κ = 0.37) for pleural line classification (homogeneous (n = 5), non-homogeneous (n = 14), discrepancy (n = 8) [4 in Control, 2 in CPE, 2 in NCAIS]). Of the 14 clips classified as non-homogeneous, poor inter-rater agreement (κ = 0.32, 95% CI 0.12–0.52) was observed for the subclassification of (irregular (n = 5), fragmented (n = 4), discrepancies (n = 5) [1 in Control, 2 in CPE, 2 in NCAIS]). Reviewer agreement for subpleural field classification was virtually non-existent (κ = − 0.06), with horizontal fields noted in 6 clips, vertical fields in 12 clips and discrepancies in 9 clips [3 in Control, 4 in CPE, 2 in NCAIS]).

#### Association between clip interpretation and underlying disease (Table 3)

**Table 3:** Comprehensive analysis of associations between disease categories and ultrasound image classifications^a^

Mode	Category	CPE	NCAIS	Normal	Total	Fisher’s Exact Test *p*-value
B-Mode	Homogenous Pleural Line	6	0	10	*16*	< 0.0001
	Non-Homogenous Pleural Line	2	16	2	*20*	
	*Total*	*8*	*18*	*10*	*36*	
M-Mode	Homogenous Pleural Line	16	0	20	*36*	< 0.0001
	Non-Homogenous Pleural Line	0	32	0	*32*	
	*Total*	*16*	*32*	*20*	*68*	
	Horizontal Subpleural Field	4	8	20	*32*	< 0.0001
	Vertical Subpleural Field	12	24	0	*36*	
	*Total*	*16*	*32*	*20*	*68*	

_*CPE* Chronic Pulmonary Embolism, *NCAIS* Non-Cardiac Associated Interstitial Syndrome, *B-Mode* Brightness Mode ultrasound, *M-Mode* Motion Mode ultrasound_

^a^The totals represent the combined results from both reviewers for each category

We examined the association between the controls and disease categories (CPE, NCAIS) and homogeneity (Homogenous, Non-Homogenous) using Fisher’s exact test (Table 3).

Of the 18 HFLUT B-mode clips, 6 were from control dogs, 4 were from dogs diagnosed with CPE, and 8 were from dogs with NCAIS. The 8 homogeneous pleural lines were either associated with control dogs (5/8) or dogs with CPE (3/8). The 9 non-homogeneous pleural lines were all from dogs with NCAIS (8/9) except one dog with CPE (1/9).

Of the 34 HFLUT M-mode clips, 10 were from control dogs, 8 were from dogs with CPE, and 16 were from dogs with NCAIS. The 18 pleural lines classified as homogeneous by the reviewers were either associated with control dogs (10/18) or CPE (8/18). The 16 non-homogeneous pleural lines were all identified in dogs with NCAIS. The 16 horizontal subpleural fields were most commonly associated with control dogs (10/16) and ZOIs of dogs with CPE (2/16, ZOIs: left caudodorsal, right cranioventral) and NCAIS (4/16; ZOIs: right cranioventral, left mediolateral) without evidence of vertical artifacts (i.e., lung fields without evidence of pulmonary parenchymal disease) in the ZOI evaluated. The 16 clips classified as having vertical subpleural fields were most commonly linked to either CPE (6/16) or NCAIS (10/16).

The Fisher’s Exact tests revealed significant associations between disease categories and homogeneity in both B-Mode and M-Mode analyses (*p* < 0.0001) when using the HFLUT. However, it is essential to note that Fisher’s exact test does not account for the variability introduced by having two observers in the study. This observer effect can introduce bias or variability, potentially influencing the results. Therefore, the significant findings should be interpreted with caution. Additionally, due to low interobserver agreement observed for clips obtained with the CUT, no comparison of clip interpretation with underlying disease was evaluated. A summary of the interobserver agreements can be found in Table 4 and a summary of the ZOIs for each transducer can be found in Supplemental Tables 1–2.

**Table 4:** Comparison of inter-rater agreement between M-mode and B-mode ultrasonography using high frequency linear ultrasound transducer (HFLUT) and curvilinear ultrasound transducer (CUT) represented by the kappa statistic (95% CI)

Ultrasound characteristic	HFLUT		CUT	
	B-Mode (n = 18)	M-Mode (n = 34)	B-Mode (n = 9)	M-Mode (n = 27)
Pleural Line Homogeneity	0.89 (0.68–1.00)	1.00 (1.00–1.00)	0.34 (− 0.25–0.94)	0.37 (0.05–0.69)
Primary Pleural Line Characteristic	0.72 (0.44–0.99)	0.90 (0.78–1.00)	0.44 (− 0.02–0.89)	0.32 (0.12–0.52)
Subpleural Field Characteristics	–	0.88 (0.72–1.00)	–	− 0.06 (− 0.41–0.28)

Kappa values range from − 1 to 1, with higher values indicating better agreement

– = not applicable

## Discussion

Our study demonstrated better inter-rater agreement for HFLUT over CUT in both B-mode and M-mode imaging for pleural line and subpleural field characterization in dogs without evidence of pulmonary parenchymal disease and dyspneic dogs. Specifically, M-mode and B-mode clips captured with HFLUT exhibited strong to near perfect agreement for pleural line abnormalities interpretation using our classification system. This suggests that a HFLUT produces images that can be more consistently described and interpreted, which may result in improved diagnostic accuracy compared to a CUT. These findings align with existing human literature indicating that higher frequency linear transducers provide enhanced visualization of superficial structures and pleura, coupled with superior axial and lateral resolution [22].

Moreover, while B-mode imaging primarily assesses anatomical structures and tissue characteristics, M-mode imaging serves as a supportive modality, providing real-time information regarding motion and dynamics [1, 15, 27, 31, 32]. In human medicine, M-mode has been previously validated for the detection of various thoracic abnormalities, including pneumothorax, pleural effusion, and hydropneumothorax, and has also been described for the evaluation of the diaphragm for paralysis and ventilator weaning [22, 30, 31]. Comparatively, in veterinary medicine, M-mode has been largely restricted to the diagnosis of pneumothorax, although clinical studies to support its use are lacking [6, 25]. In our study, we found that there was greater inter-observer agreement amongst reviewers when reviewing the pleural line in M-mode ultrasonographic profile using the HFLUT for dogs with SLIS compared to B-mode using either the HFLUT or the CUT. The one-dimensional representation of motion over time in M-mode likely allows for the identification of specific fields, such as changes in pleural line movement and the presence of vertical artifacts, which are indicative of AIS. These fields were likely easier to recognize and less subjective compared to the real-time, two-dimensional images of B-mode ultrasonography. Consequently, this reduces variability between observers and enhances the consistency and reliability of the assessments, making M-mode a valuable tool in the evaluation of SLIS in veterinary lung POCUS. Further studies enrolling a larger population would be valuable to further evaluate differences between M-mode and B-mode ultrasound to characterize the pleural line.

In current literature, the term ‘vertical artifact’ encompasses a range of phenomena observable on lung ultrasound, the specific criteria for defining and categorizing these artifacts, including B-lines, vary across the literature [9, 11, 21, 22]. In our study, we opted to use the broader term ‘vertical artifact’, in lieu of “B-line”, to reflect the potential for variability in appearance caused by differences in pathology (e.g., fibrosis versus fluid) and technical factors (e.g., machine settings). Thus, this terminology reduces the risk of misclassification and prioritizes inter-rater variability rather than the precise categorization of the artifact’s origin.

For subpleural field characterization, HFLUT’s M-mode also appears to showcase the subpleural field more clearly compared to CUT’s M-mode and B-mode. Furthermore, the strong inter-rater agreement with HFLUT suggests that it might offer a more reliable method of assessing pleural line integrity and the nature of the subpleural fields, which may subsequently improve diagnostic accuracy, although further research is required to support this hypothesis. In light of this, further research to evaluate the pleural line, subpleural fields, and their combinations in dogs presenting in respiratory distress using a HFLUT and a combination of B- and M-mode ultrasonography is warranted.

In our study, we adopted a standardized language derived from human medicine to describe pleural line abnormalities. Recognizing the absence of a consensus for such descriptions in veterinary medicine, we either adapted or formulated definitions from Fischer et al. [15] to establish fundamental characteristics of pleural line abnormalities. While human medicine offers further characterization of pleural lines, applying these inconsistently to veterinary patients poses challenges due to anatomical and breed-associated nuances. Therefore, our approach aimed to tailor definitions to better suit the veterinary context in the hope of creating a more uniform research approach, and diagnostic characterization of AIS.

Using M-mode with a HFLUT in our study, we consistently observed associations between a homogeneous pleural line in either control dogs or those with cardiogenic pulmonary edema (CPE), while a non-homogeneous pleural line was associated with non-cardiogenic alveolar interstitial syndrome (NCAIS). Similarly, the vertical subpleural field identified with HFLUT was associated with either CPE or NCAIS in 16 out of 18 (89%) of the reviewed clips with only two clips of 18 clips (11%), having a discrepancy amongst the reviewers. These findings are similar to those of Singh et al. [32], where in people, the combination of a continuous pleural line and a vertical subpleural field was associated with CPE, and a non-continuous, fragmented pleural line and vertical subpleural field was associated with NCAIS. Furthermore, while the horizontal subpleural field was most frequently associated with control dogs in this patient population, it was also found in several clips within the CPE and NCAIS groups as well. However, it should be noted that horizontal subpleural field identified in patients with CPE and NCAIS were observed in ZOI's without vertical artifacts, which likely explains the discrepancies. Further research is needed to confirm the association identified in this pilot study.

Several limitations should be considered when interpreting the findings of this study. There were a limited number of clips from only nine dogs. While a sample size calculation was performed for statistical purposes, future research with larger sample sizes is warranted to validate our findings and elucidate factors influencing interobserver agreement in ultrasound imaging interpretation (e.g., breeds with various conformations, sizes, sonographer and clip reviewers’ level of experience etc.). For comparison, the study by Fatima et al. (2022) demonstrated higher inter-rater reliability with six human operators analyzing 1035 lung ultrasound videos in COVID-19 patients [14]. However, in veterinary medicine, the use of two expert reviewers allows for consistent, high-quality interpretations essential in an emerging field like veterinary LUS. This approach emphasizes expertise over quantity, reducing variability that could arise with less-experienced reviewers. Additionally, the selection of two reviewers was supported by a power analysis based on other veterinary studies [10, 24, 25, 36, 37], which indicated this would yield statistically meaningful results. Adding more reviewers would require substantial training and resources, potentially overcomplicating the study without improving the reliability of findings. However, the authors acknowledge that future studies could benefit from incorporating a broader pool of evaluators with different levels of experience to further assess consistency across ultrasound assessments, particularly in veterinary settings. This study could also be impacted by variability in imaging technique, although only one investigator (KG) performed all ultrasonographic examinations of the dogs enrolled in the study. Furthermore, the clips attained included videos, which diminished the impact of transducer handling or patient movement (e.g., oblique angling of the transducer during examination) by capturing the lung movement or sliding at various angles throughout the examination. Next, bias on the part of the reviewers to favor one transducer over another could not be blinded as the appearance of the image easily differentiates HFLUT from CUT B-mode clips. Also, the focal point was not specifically adjusted to the pleural line, which may have influenced the findings. However, given that all reviewers assessed the same clips with the same focal position, this limitation is unlikely to have impacted inter-rater variability.

Furthermore, to provide a more robust analysis, a linear mixed model (LMM) would be more appropriate for the statistical analysis of the associations between diseases and clip classifications. LMM can adjust for both fixed effects (e.g., disease category) and random effects (e.g., observer variability). Moreover, our methodology did not involve quantitative counting of vertical artifacts but rather focused on qualitative characterization of pleural and subpleural fields to provide a robust assessment of lung pathology. However, implementing an LMM requires a larger sample size, which was not available in the current study. Future research should aim to collect a larger sample size to enable the use of LMM or similar approaches for a more accurate and reliable analysis of the data, accounting for observer variability.

Finally, an advancing frontier in LUS is the integration of algorithmic and computer-aided diagnostic (CAD) systems to overcome the limitations inherent in manual interpretation. Modern developments suggest that a standardized CAD approach could greatly enhance the identification and quantification of pleural line and subpleural features, increasing consistency across clinicians. Corradi et al. [7] demonstrated that algorithmic LUS, utilizing gray-level co-occurrence matrix (GLCM) and second-order texture analysis, effectively reduces observer variability by emphasizing characteristic features of various respiratory pathologies. This technology could facilitate more uniform LUS assessments, achieving consistent diagnostic scores and precise monitoring metrics, independent of an operator’s expertise. Additionally, CAD tools can streamline LUS assessments by automating pleural line and subpleural feature quantification, thus minimizing observer variability and supporting robust, reproducible scoring frameworks in both research and clinical applications [14].

## Conclusions

Our study demonstrated that a HFLUT achieved better inter-observer agreement compared to a CUT in both B-mode and M-mode imaging for pleural line and subpleural field characterization, suggesting a HFLUT produces more consistently interpretable images, which may improve diagnostic accuracy in the clinical setting. Furthermore, homogeneous pleural lines were consistently identified in control dogs and those with CPE using both transducers, while non-homogeneous pleural lines, particularly irregular or fragmented ones, were associated with NCAIS and were more frequently detected with a HFLUT, indicating its sensitivity to subtle pleural abnormalities. Horizontal subpleural fields were exclusive to control dogs using a HFLUT, whereas vertical fields were linked to CPE and NCAIS, reinforcing the diagnostic value of subpleural field evaluation and the utility of M-mode in canine lung POCUS. Further studies are needed to determine if pleural line and subpleural field characteristics can reliably differentiate the etiology of vertical artifacts in small animals presented in respiratory distress when images are obtained with a HFLUT in B- or M-mode ultrasonography.

## Supplementary Information

Additional file 1.

## Data Availability

The datasets generated and/or analyzed during the current study are available from the corresponding author on reasonable request. Supplementary information, including figures and tables, is provided in the manuscript and its supplementary files.

## Abbreviations

ADI: Advanced diagnostic imaging
AIS: Alveolar interstitial syndrome
ARDS: Acute Respiratory Distress Syndrome
B-mode: Brightness mode
CPE: Cardiogenic pulmonary edema
CT: Computed tomography
CUT: Curvilinear transducer
EC: Echocardiography
HPL: Homogenous Pleural Line
HSP: Horizontal Subpleural field
NHPL: Non-Homogenous Pleural Line
LA:Ao ratio: Left atrial-to-aorta ratio
HFLUT: High-frequency linear ultrasound transducer
M-mode: Motion mode
NCAIS: Non-cardiogenic alveolar-interstitial syndrome
POCUS: Point-of-care ultrasound
SLIS: Sonographic lung interstitial syndrome
TXR: Thoracic radiographs
VSP: Vertical Subpleural field
ZOI: Zone of interest
