# Supplementary material for: Inter-rater agreement and characterization of pleural line and subpleural fields in canine lung ultrasound: a comparative pilot study between high-frequency linear and curvilinear transducers using B- and M-mode ultrasonographic profiles
Source: Ultrasound J. 2025 Jan 13;17:3. doi: 10.1186/s13089-025-00401-z (PMC11729591; doi:10.1186/s13089-025-00401-z)
Supplement: Supplementary file 1 — Additional file 1. [file 13089_2025_401_MOESM1_ESM.docx]

| **Supplemental Table 1. Detailed breakdown of the HFLUT B- and M-Mode clip classification and discrepancies based on ZOIs** | | | | | | |
| --- | --- | --- | --- | --- | --- | --- |
| **ZOI** | **Disease Group** | **HFLUT B-Mode Clips** | | **HFLUT M-Mode Clips** | | |
|  |  | **Pleural Line** | **Pleural Line Discrepancy** | **Pleural Line** | **Pleural Line Discrepancy** | **Subpleural Field Discrepancy** |
| Left Caudodorsal | Control | 1 | 1 | 2 | 0 | 0 |
|  | CPE | 1 | 0 | 1 | 0 | 0 |
|  | NCAIS | 1 | 0 | 2 | 0 | 0 |
| Right Caudodorsal | Control | 1 | 0 | 1 | 0 | 0 |
|  | CPE | 1 | 0 | 3 | 0 | 0 |
|  | NCAIS | 1 | 0 | 2 | 0 | 1 |
| Left Perihilar | Control | 0 | 0 | 0 | 0 | 0 |
|  | CPE | 2 | 0 | 4 | 0 | 0 |
|  | NCAIS | 1 | 0 | 1 | 0 | 0 |
| Right Perihilar | Control | 1 | 0 | 2 | 0 | 0 |
|  | CPE | 1 | 0 | 1 | 0 | 0 |
|  | NCAIS | 0 | 0 | 0 | 0 | 0 |
| Left Cranioventral | Control | 1 | 0 | 2 | 0 | 0 |
|  | CPE | 1 | 0 | 2 | 0 | 0 |
|  | NCAIS | 0 | 0 | 0 | 0 | 0 |
| Right Cranioventral | Control | 1 | 0 | 2 | 0 | 0 |
|  | CPE | 0 | 0 | 0 | 0 | 0 |
|  | NCAIS | 1 | 0 | 3 | 0 | 0 |
| Left Medioventral | Control | 1 | 0 | 1 | 0 | 0 |
|  | CPE | 0 | 0 | 0 | 0 | 0 |
|  | NCAIS | 0 | 0 | 1 | 0 | 0 |
| Right Medioventral | Control | 1 | 0 | 2 | 0 | 0 |
|  | CPE | 0 | 0 | 0 | 0 | 0 |
|  | NCAIS | 1 | 0 | 2 | 0 | 1 |
| Total |  | 18 | 1 | 34 | 0 | 2 |
| _1D: One Dimensional, B-mode: Brightness mode, CPE: Cardiogenic Pulmonary Edema, CUT: Curvilinear Ultrasound Transducer, HFLUT: High Frequency Linear Ultrasound Transducer, M-mode: Motion mode, NCAIS: Non-Cardiogenic Alveolar-Interstitial Syndrome, ZOI: Zone of Interest_ | | | | | | |

| **Supplemental Table 2. Detailed breakdown of the CUT B- and M-Mode clip classification and discrepancies based on ZOIs** | | | | | | |
| --- | --- | --- | --- | --- | --- | --- |
| **ZOI** | **Disease Group** | **CUT B-Mode Clips** | | **CUT M-Mode Clips** | | |
|  |  | **Pleural Line** | **Pleural Line Discrepancy** | **Pleural Line** | **Pleural Line Discrepancy** | **Subpleural Field Discrepancy** |
| Left Caudodorsal | Control | 1 | 0 | 2 | 1 | **1** |
|  | **CPE** | **1** | **0** | **2** | **0** | **0** |
|  | NCAIS | 0 | 1 | 0 | 0 | **0** |
| Right Caudodorsal | Control | 0 | 0 | 0 | 0 | **0** |
|  | **CPE** | **1** | **0** | **2** | **2** | **2** |
|  | NCAIS | 1 | 1 | 2 | 0 | **0** |
| Left Perihilar | Control | 1 | 0 | 1 | 1 | **1** |
|  | **CPE** | **0** | **0** | **0** | **0** | **0** |
|  | NCAIS | 0 | 0 | 0 | 0 | **0** |
| Right Perihilar | Control | 0 | 0 | 1 | 1 | **0** |
|  | **CPE** | **1** | **0** | **2** | **0** | **1** |
|  | NCAIS | 1 | 0 | 2 | 1 | **1** |
| Left Cranioventral | Control | 1 | 0 | 2 | 1 | **1** |
|  | **CPE** | **0** | **0** | **0** | **0** | **0** |
|  | NCAIS | 0 | 0 | 0 | 0 | **1** |
| Right Cranioventral | Control | 0 | 0 | 1 | 0 | **0** |
|  | **CPE** | **0** | **0** | **0** | **0** | **0** |
|  | NCAIS | 0 | 1 | 1 | 0 | **0** |
| Left Medioventral | Control | 0 | 0 | 2 | 0 | **0** |
|  | **CPE** | **0** | **0** | **0** | **0** | **0** |
|  | NCAIS | 0 | 0 | 2 | 0 | **0** |
| Right Medioventral | Control | 0 | 0 | 1 | 0 | **0** |
|  | **CPE** | **0** | **0** | **2** | **0** | **1** |
|  | NCAIS | 1 | 0 | 2 | 1 | **0** |
| **Total** |  | **9** | **3** | **27** | **8** | 9 |
| _1D: One Dimensional, B-mode: Brightness mode, CPE: Cardiogenic Pulmonary Edema, CUT: Curvilinear Ultrasound Transducer, HFLUT: High Frequency Linear Ultrasound Transducer, M-mode: Motion mode, NCAIS: Non-Cardiogenic Alveolar-Interstitial Syndrome, ZOI: Zone of Interest_ | | | | | | |
